# From Interpersonal Insecurity to Disordered Eating: The Mediating Pathway of Appearance-Based Rejection Sensitivity

**DOI:** 10.3390/bs16020307

**Published:** 2026-02-22

**Authors:** Liang Zhang, Yang Zeng, Yanqiang Tao, Xiangping Liu, Shujian Wang

**Affiliations:** 1 College Students’ Mental Health Education Center, Northeast Agricultural University, Harbin 150030, China; zhangliangpsy@neau.edu.cn; 2 Faculty of Psychology, Beijing Normal University, Beijing 100875, China; 202211998373@mail.bnu.edu.cn (Y.Z.); 89034@bnu.edu.cn (X.L.); 3 Beijing Key Laboratory of Applied Experimental Psychology, National Demonstration Center for Experimental Psychology Education, Beijing 100875, China; 4 Shanghai Key Laboratory of Mental Health and Psychological Crisis Intervention, School of Psychology and Cognitive Science, East China Normal University, Shanghai 200062, China; yqtao@psy.ecnu.edu.cn

**Keywords:** attachment anxiety, appearance-based rejection sensitivity, anorexia nervosa, gender difference, eating disorder

## Abstract

Eating disorders among adolescents have emerged as a significant global public health concern, with attachment anxiety identified as a critical risk factor for anorexia nervosa (AN) symptoms. Individuals with attachment anxiety often exhibit heightened sensitivity to others’ perceptions and an intense fear of rejection, which may exacerbate their vulnerability to body image concerns. This study investigates the mediating role of appearance-based rejection sensitivity (ARS) and the moderating effect of sex in the relationship between attachment anxiety and AN symptoms among Chinese college students. A total of 826 participants aged 16–25 (M = 18.95, SD = 1.08, 60% females) completed online surveys using three validated scales: the Revised Adult Attachment Scale (RAAS) to assess attachment anxiety, the Appearance-based Rejection Sensitivity Scale (ARSS) to measure sensitivity to rejection related to physical appearance, and the Eating Disorders Inventory (EDI) to evaluate AN symptoms. The results reveal that there was no significant difference in attachment anxiety between males and females, while females experienced significantly higher levels of ARS and severity of AN symptoms than males. Attachment anxiety is positively correlated with AN symptoms, with ARS mediating this association. Sex further moderates the relationship between ARS and AN symptoms, with stronger effects observed in females. Current findings suggest that AN symptoms are closely associated with contemporaneous attachment anxiety, with passive sensitivity to potential rejection acting as a mediating factor. This underscores the importance of addressing attachment styles and communication patterns in interventions targeting adolescent AN symptoms, particularly in females.

## 1. Introduction

Anorexia nervosa (AN) is a complex eating disorder characterized by significant disturbances in eating patterns and a self-evaluation system excessively dominated by body shape and weight. While global prevalence among adolescents has surged, the specific vulnerability marker for AN symptoms remains a subject of intense investigation (López-Gil et al., 2023). Individuals prone to AN symptoms often perceive their bodies through the “gaze of others,” lacking internal proprioceptive awareness (Monteleone et al., 2017). Mounting evidence suggests that attachment patterns, particularly attachment anxiety, play a critical role in this pathology. Attachment anxiety involves an excessive need for approval and an intense fear of interpersonal rejection. Despite established correlations between attachment insecurity and eating disorders (Chris Fraley et al., 2006; Klein et al., 2022), the mediating pathways—specifically how interpersonal fears translate into body-related behaviors—require further clarification. To address this gap, the current study investigates whether appearance-based rejection sensitivity (ARS) mediates the relationship between attachment anxiety and AN symptoms, while also exploring potential sex differences in this process.

### 1.1. Attachment Anxiety and Anorexia Nervosa Symptoms

Attachment anxiety refers to an excessive need for others’ approval and a fear of interpersonal rejection (Dakanalis et al., 2014), coupled with low self-confidence (Mikulincer & Shaver, 2007). In line with this definition, being attuned to others’ perspectives and seeking their approval are primary features that correspond with AN symptoms. Empirical studies support a positive association between attachment anxiety and AN across various ages, sexes, and cultures. For instance, one study demonstrated a positive correlation between attachment anxiety and AN symptoms among Italian adults (Dakanalis et al., 2014). Similarly, anxious attachment style significantly co-varied with AN symptoms among Germans, spanning ages 14 to 92 years (Klein et al., 2022). A systematic review further indicated that attachment insecurity may confer a risk for developing AN symptoms (Tasca & Balfour, 2014). Therefore, based on previous evidence, we hypothesize that attachment anxiety is positively associated with AN symptoms.

### 1.2. Appearance-Based Rejection Sensitivity as a Mediator

Appearance-based rejection sensitivity (ARS) is defined as anxious concerns and expectations about being rejected based on one’s physical attractiveness, such as shape and figure (Park, 2007). ARS consists of two components: anxiety about rejection (the emotional component) and attributing rejection to appearance (the cognitive component). These components exacerbate each other, forming a vicious cycle that triggers negative consequences such as lower self-esteem and feelings of loneliness (Park, 2007).

According to attachment theory, ARS can be seen as a protective way of responding to a defensive environment, characterized by heightened apprehension toward potential rejection within relationships (De Paoli et al., 2017b). From a social interaction perspective, adolescents exhibiting attachment anxiety may frequently confront rejection and abandonment, which is theoretically rooted in inconsistent caregiving experiences during childhood (Bowlby & McKie, 2019). In the context of emerging adulthood, this manifests as a stable relational disposition characterized by a heightened need for approval and fear of rejection (DeWall et al., 2012; Manning et al., 2017; Yue & Stefanone, 2022). On the other hand, they tend to harbor a strong inclination towards intimacy and closeness (Toma, 2022). Consequently, such individuals are prone to hypersensitivity toward rejection cues (De Paoli et al., 2017b). From a cognitive perspective, rejection is construed as a negative stimulus, thereby instigating heightened vigilance among individuals with attachment anxiety towards potential relational threats (De Paoli et al., 2017a). This heightened vigilance predisposes them to interpret neutral or ambiguous cues as potential precursors of rejection. Therefore, we hypothesized that attachment anxiety is positively related to ARS.

Meanwhile, individuals with high ARS view their appearance as a key factor in inducing rejection and are more vulnerable to negative comments about their appearance, often excessively preoccupying themselves with physical characteristics (De Paoli et al., 2017a; Park, 2007). Considering modern societal views of a slender body as attractive (Swami et al., 2010), individuals high in ARS may feel a stronger motivation to improve their body image, leading to disordered eating behaviors. ARS has been found to positively predict body dysmorphic disorder symptoms, social anxiety, and a range of social dysfunctions (Lavell et al., 2014; Linardon et al., 2017). Park’s studies on ARS in adolescents revealed that ARS significantly jeopardizes adolescent mental health, with high-ARS adolescents more likely to undergo cosmetic surgery to improve their appearance (Park, 2007; Park et al., 2009). Pitiruţ et al. (2024) found that ARS can increase the likelihood of dysmorphic concerns. Thus, individuals with high ARS are more likely to develop AN symptoms to help themselves lose weight in order to maintain an attractive appearance (as perceived by themselves) thereby reducing appearance anxiety. Given these reasons, it is plausible that ARS mediates the association between attachment anxiety and AN symptoms.

### 1.3. Sex as a Moderator

Although attachment anxiety might be related to AN symptoms via ARS, not all individuals with high ARS will display anorexic behaviors. Females are more likely than males to report body checking and binge eating (Striegel-Moore et al., 2009). Schmidt and Martin (2019) found that the effect of appearance teasing on mental health was stronger and mediated by ARS in females compared to males. According to Cognitive Social Learning Theory (CSLT), the behaviors of both males and females are shaped by different reinforcements, punishments, and stereotypes established by social norms, which affect self-regulation and psychological expectations (Hyde, 2014). Specifically, females are more likely than males to overestimate the importance of physical appearance and have stronger motivations to change their body image. Research shows that females are more prone to internalizing societal beauty ideals, contributing to both body dissatisfaction and the desire to alter their appearance. Exposure to idealized beauty standards on social media platforms like Instagram significantly increases body dissatisfaction in females, leading them to engage in behaviors such as dieting or exercising to meet these standards. This effect is more pronounced in females, while males tend to report lower levels of body dissatisfaction under similar conditions (Fioravanti et al., 2022). Additionally, sex differences in coping strategies indicate that female adolescents are more likely than male adolescents to struggle with body dissatisfaction and use fewer active strategies to mitigate these negative effects (Mahon & Hevey, 2021). Consistent with CSLT, a narrative review revealed that sex influences AN risk through body image and self-esteem based on clinical and community samples (Breton et al., 2023). Based on CSLT and previous evidence, we hypothesize that sex moderates the relationship between ARS and AN symptoms.

### 1.4. The Current Study

**Figure 1:**
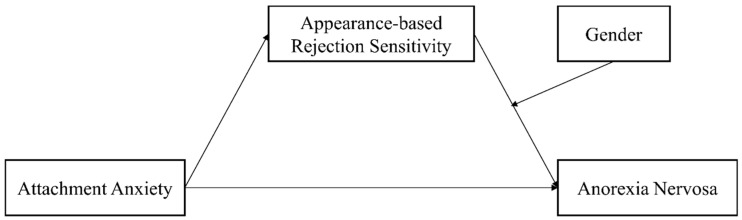
The proposed moderated mediation model in the current study.

The current study aims to test whether attachment anxiety is associated with AN symptoms via ARS and to investigate the moderating role of sex. Based on the literature review, we propose the following hypotheses: (1) Attachment anxiety is positively related to AN symptoms. (2) ARS mediates the relationship between attachment anxiety and AN symptoms. (3) Sex moderates the relationship between ARS and AN symptoms. Specifically, high ARS in females is more likely to be positively correlated with AN symptoms, whereas this correlation is lower for males. The proposed model is shown in Figure 1.

## 2. Method

### 2.1. Participants and Procedure

Data were collected through an online questionnaire administered at Northeast Agricultural University in October 2024. University staff members distributed the questionnaire link to student chat groups, inviting students to participate. A total of 888 responses were obtained. Prior to participation, all individuals provided electronic informed consent. Invalid questionnaires were eliminated based on specific criteria, including: (1) excessively short response times; (2) incorrect responses to attention detection questions; and (3) presence of incomplete questions. Following this screening process, 826 valid questionnaires remained for analysis, with participant ages ranging from 16 to 25 years (M = 17.95, SD = 1.08, 60% females). To determine the adequacy of the sample size for the proposed moderated mediation model, we conducted a power analysis using a simulation-based approach (Xu et al., 2024). Setting the type I error rate at α = 0.05 and assuming a moderate effect size (f^2^ = 0.15) for the moderated mediation effect, a Monte Carlo simulation with 826 participants yielded a statistical power of 0.91. This exceeds the conventional threshold of 0.80, indicating sufficient sensitivity to detect the hypothesized effects. The inclusion of participants aged 16 and 17 (2 participants aged 16, 22 participants aged 17) is consistent with the presence of younger-than-average students in the Chinese higher education system who have undergone early school enrollment. Regarding the ethical procedure for minors, the Ethics Committee of Beijing Normal University approved the electronic informed consent process for all enrolled university students, as the research was classified as minimal risk. The Ethics Committee of Beijing Normal University approved the current research (IRB number: 202409290170).

### 2.2. Measures

#### 2.2.1. Demographic

To control for potential confounding effects, we collected relevant demographic information through self-reported, including age, sex, subjective economic status (SES), parental education level, relationship status, and whether they had previous romantic experiences. These variables were selected based on theoretical and empirical evidence linking them to attachment patterns, appearance concerns, or eating behaviors (Davies & Cummings, 1994; Laus et al., 2018; Liu et al., 2023; West et al., 2019).

#### 2.2.2. Revised Adult Attachment Scale (RAAS)

Attachment anxiety was evaluated using the Revised Adult Attachment Scale (RAAS), originally devised by Collins (1996) and adapted into Chinese by Wu et al. (2004). The RAAS consists of 18 5-point items distributed across three subscales: closeness, dependence, and anxiety. The anxiety subscale gauges individuals’ concerns about potential rejection or abandonment by others. In our investigation, we employed the anxiety subscale to gauge participants’ attachment anxiety, with higher scores indicating greater levels of attachment anxiety. The anxiety subscale of the RAAS demonstrated satisfactory reliability in this study (Cronbach’s α = 0.79).

#### 2.2.3. Appearance-Based Rejection Sensitivity Scale (ARSS)

The assessment of appearance-based rejection sensitivity utilized the Appearance-based Rejection Sensitivity Scale (ARSS), developed by Park (2007) and adapted into Chinese by Deng et al. (2018). This scale comprises 15 scenarios, each accompanied by two questions: one assessing anxiety regarding rejection (rated on a 6-point scale ranging from 1 = not worried at all to 6 = very worried), and the other evaluating attributions concerning rejection based on one’s appearance (rated on a 6-point scale ranging from 1 = not likely at all to 6 = very likely). The scores from both questions in each scenario were multiplied, and the mean value across all 15 scenarios constituted the individual’s ARSS score. Higher scores indicate greater appearance-based rejection sensitivity in participants. In the current study, the ARSS demonstrated strong reliability, with a Cronbach’s α coefficient of 0.91.

#### 2.2.4. Eating Disorders Inventory (EDI)

Participants’ AN symptoms were measured using the Eating Disorders Inventory (EDI), originally developed by Garner et al. (1983) and later revised into a simplified Chinese version by Zhang and Kong (2004). The EDI comprises eight dimensions: drive for thinness, body dissatisfaction, bulimia, ineffectiveness, perfectionism, interpersonal distrust, interoceptive awareness, and maturity fears. The first three dimensions are central to the pathology of anorexia nervosa and bulimia nervosa, while the remaining dimensions assess psychopathology that is commonly associated with, but not unique to, eating disorders (Pike & Mizushima, 2005). Since this study focused on the effects of attachment anxiety on core AN symptoms, we selected two subscales, drive for thinness and body dissatisfaction, to measure participants’ AN symptoms. The mean scores of the 16 items from these two subscales represent the severity of the individual’s AN symptoms. In this study, the Cronbach’s α values for the two selected subscales were 0.85 and 0.87, respectively.

### 2.3. Data Analysis

All data analyses were performed in R version 4.2.2. First, Harman’s single-factor test was used to test for common method bias. The factor analysis extracted 4 factors (eigenvalue > 1), with the most important factor accounting for 21.3% (less than 40%) of the variance, suggesting no significant common method variance. Second, a multicollinearity analysis was conducted to check whether the predictor variables were related to each other. The variance inflation factor (VIF) of all predictors were 1.05~2.85 (less than 5), reflecting no severe multicollinearity. Next, the kurtosis and skewness values were used to test whether variables had a normal distribution. Byrne (2016) argued that data is considered to be normal if skewness is between −2 to +2 and kurtosis is between −7 to +7. According to this criterion, the variables were in line with normal distribution. The R package lavaan was used to examine the mediating effect and moderated mediating effect. All predictors were centered prior to analysis.

## 3. Results

### 3.1. Preliminary Analyses

The detailed demographic information and chi-square test results between males and females are shown in Table 1.

An independent sample *t*-test was performed to explore the sex differences in main variables. Table 2 presents the descriptive information and t-test results of attachment anxiety, ARS, and AN symptoms. The *t*-test results showed that female participants exhibited significantly higher ARS (t = 2.83, *p* < 0.01, Cohen’s d = 0.20) and more severe AN symptoms (t = 10.74, *p* < 0.001, Cohen’s d = 0.76) than males.

The Pearson correlations are shown in Table 3. The results indicated that attachment anxiety was significantly positive related to ARS (r = 0.26, *p* < 0.001) and AN symptoms (r = 0.40, *p* < 0.001), while ARS was significantly positive related to AN symptoms (r = 0.40, *p* < 0.001).

### 3.2. Testing the Mediating Role of ARS in the Mediation Model

As shown in the mediation model results (the left columns of Table 4), after controlling for age, whether having romantic experiences, whether in a romantic relationship, SES, father education level, and mother education level, we found that attachment anxiety was significantly and positively associated with AN symptoms (β = 0.14, SE = 0.04, *p* < 0.001). A strong positive association was also found between attachment anxiety and ARS (β = 2.72, SE = 0.22, *p* < 0.001). The correlation between ARS and AN symptoms was also significant (β = 0.06, SE = 0.01, *p* < 0.001). The indirect effect of attachment anxiety on AN symptoms was significant (β = 0.17, bootstrapped 95% CI = [0.12, 0.21]). These results indicated that the effect of AA on AN symptoms was partially mediated by ARS.

### 3.3. Testing the Moderating Role of Sex in the Moderated Mediation Model

As shown in the moderated mediation model results (the right columns of Table 4), the interaction of ARS and sex was positively related to AN symptoms, and the effect size was significant (β = 0.03, SE = 0.01, *p* < 0.05). This result indicated that sex moderates the relationship between ARS and AN symptoms. The bootstrapped test (5000 iterations) also showed that the difference between two conditional indirect effects was 0.07, with a 95% confidence interval of [0.01, 0.13]. These results suggested that the indirect effect of attachment anxiety on AN symptoms was stronger among females compared to males, although it was significant for both sexes.

**Figure 2:**
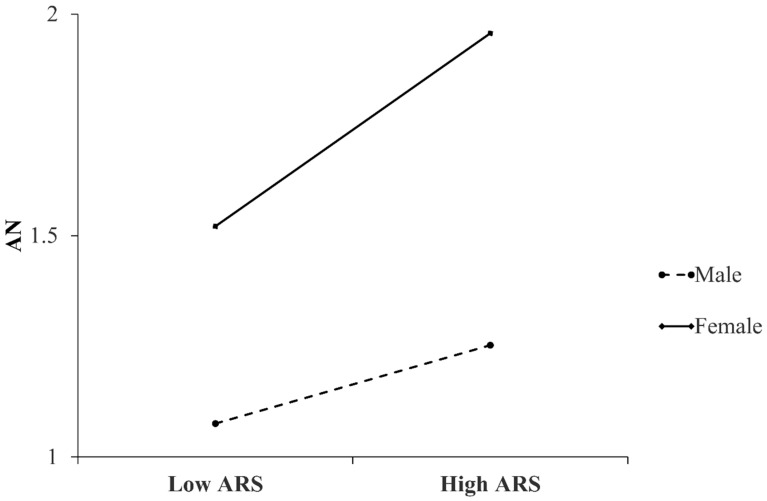
Simple slopes plot of the conditional effects of ARS on AN symptoms with sex as a moderator. Note: ARS = Appearance-based Rejection Sensitivity; AN = Anorexia Nervosa symptoms.

The simple slope test was conducted better to understand the interaction effects of ARS and sex. As shown in Figure 2, the predictive relationship between ARS and AN symptoms among females (simple slope = 0.07, SE = 0.01, t = 9.22, *p* < 0.001) was stronger than males (simple slope = 0.04, SE = 0.01, t = 4.86, *p* < 0.001). Taking the above results into account, the Hypothesis 3 was supported.

### 3.4. Sensitivity Analysis

To examine the extent to which the control variables included in the model influence the current results, we constructed a model that excludes these control variables. Results indicated that the inclusion of these covariates did not change the direction or statistical significance of any of the primary hypothesized paths (the direct effect of attachment anxiety on AN symptoms = 0.13, SE = 0.04, t = 3.26, *p* < 0.01; the mediating effect of ARS = 0.16, SE = 0.02, t = 6.48, *p* < 0.001; the moderating effect of sex = 0.03, SE = 0.003, t = 7.51, *p* < 0.001).

## 4. Discussion

In light of the increasing prevalence of AN symptoms among adolescents and adults, this study examined the relationship between attachment anxiety and AN symptoms among Chinese college students. Additionally, it explored the mediating role of ARS and the moderating effect of sex in the association between attachment anxiety and AN. The findings enhance our understanding of how attachment anxiety is associated with AN symptoms, approaching the issue from a perspective centered on healthy individuals rather than a purely pathological framework. Furthermore, the results provide empirical evidence that can inform interventions for AN symptoms amidst rising societal pressures.

### 4.1. The Relationship Between Attachment Anxiety, Anorexia Nervosa Symptoms, and the Mediating Role of ARS

The hypothesis that attachment anxiety predicts AN symptoms via ARS was supported, consistent with previous research (De Paoli et al., 2017a; Lavell et al., 2014; Linardon et al., 2017; Park, 2007). Within the framework of attachment theory, insecure attachment can affect individuals’ social functioning, relational perceptions, and emotional regulation (Collins & Read, 1990). Individuals with higher levels of attachment anxiety typically exhibit a diminished sense of self-efficacy (Wei et al., 2005). They strive for external validation yet struggle with self-acceptance in various domains, including physical appearance and perceived competence (Lin, 2015). Such individuals tend to be overly sensitive to criticism and may exaggerate perceived hostility, associating to heightened anxiety in relationships and an excessive focus on others’ opinions (Gawęda et al., 2018). When this excessive concern for the views of others extends to body image, it manifests as ARS. In response to this anxiety, AN symptoms may arise (De Paoli et al., 2017b).

We can also situate ARS within a broader developmental framework that considers how early caregiving environments shape an individual’s psychological architecture. Evidence suggests that attachment anxiety and ARS share common developmental roots in adverse parenting practices (Schimmenti & Bifulco, 2015; Webb et al., 2017). Such practices may further delineate the trajectory of an individual’s disordered eating behaviors. In a recent study, researchers have integrated the effects of adverse parenting styles on disordered eating within the framework of attachment theory, suggesting that emotional neglect and overprotection may act both independently and synergistically to increase vulnerability to disordered eating (La Rosa et al., 2025).

Emotional neglect is characterized by the persistent absence of emotional availability and responsiveness to a child’s affective needs (Haworth et al., 2024). Such neglect fundamentally impairs the maturation of affect regulation, as the child receives limited scaffolding to recognize, organize, or soothe internal states (Simon et al., 2024). In the absence of effective internal regulation, individuals may develop a heightened reliance on external, tangible cues to modulate distress and navigate social environments (Wang et al., 2024). Within this context, ARS may emerge as a compensatory mechanism, where individuals utilize the “gaze of others” as a primary metric for relational safety when internal regulatory resources are underdeveloped.

Concurrently, parental overprotection and overcontrol contribute to this vulnerability through autonomy restriction. By excessively intervening or managing challenges on the child’s behalf, caregivers may inadvertently discourage independent exploration and foster a lasting sense of incompetence (Bruysters & Pilkington, 2023). This restriction of agency has been consistently linked to disordered eating; for those lacking a sense of autonomy in other life domains, food intake and body image often emerge as proximal areas where control feels tangible and accessible (Casale et al., 2025).

When early caregiving is perceived as conditional or intrusive, individuals often internalize the belief that acceptance is contingent upon fulfilling external expectations (Casale et al., 2023; dos Reis Soares et al., 2020). This externalization of self-worth creates a psychological milieu where the self-concept is externally anchored and hypersensitive to evaluative feedback. Consequently, individuals with high attachment anxiety may exhibit elevated ARS because they construe their appearance as the primary “currency” for interpersonal belonging. The current finding that ARS mediates the link to subclinical AN symptoms is consistent with linear predictive models suggesting that perceived parenting styles shape early maladaptive schemas and relational dispositions, which subsequently drive disordered eating patterns (Joshua et al., 2024).

From a developmental perspective, adult attachment styles are widely theorized to be fundamentally shaped by early caregiving experiences and the quality of primary relational bonds (Bowlby & McKie, 2019). This theoretical framework implies that insecure early attachment or adverse childhood experiences may serve as distal risk factors for the development of AN symptoms later in life. However, a critical distinction must be maintained between these developmental antecedents and the contemporaneous psychological states measured in the current study. It is important to clarify that our results specifically highlight how current internal working models of interpersonal insecurity, rather than directly assessed childhood events, contribute to AN symptoms. Because our assessment (RAAS) reflects the participants’ present relational dispositions and internal representations, the hypothesized link between early childhood history and subsequent AN symptoms remains an indirect inference in this context. Therefore, while our findings align with the broader logic of attachment theory, the developmental assumption that childhood caregiving directly predicts adult AN symptoms through these mediators should be tested more cautiously and rigorously by future researchers using longitudinal designs or direct assessments of childhood history.

Meanwhile, it is essential to distinguish the subclinical AN symptoms observed in this study from the clinical pathology of diagnosed AN. While our findings suggest that attachment anxiety and ARS are significant vulnerability markers for AN symptoms in a non-clinical sample, these results represent an elevated risk profile rather than a clinical state. In non-clinical populations, these symptoms may manifest as body dissatisfaction and a drive for thinness, whereas clinical AN involves more severe physiological and psychological impairments that were not assessed here (Pike & Mizushima, 2005).

### 4.2. The Moderating Role of Sex

While the results support Hypothesis 3—indicating that sex moderates the mediating effect of ARS between attachment anxiety and AN symptoms—the psychological magnitude of this interaction warrants careful interpretation. Although the difference between the conditional indirect effects for males and females reached statistical significance, the absolute magnitude of this difference (0.07) was modest. This suggests that while the pathway from ARS to AN symptoms is more pronounced in females, sex likely serves as a proxy for broader sociocultural and socialization processes rather than an inherent psychological driver.

The marginally stronger effect observed in females may be attributed to gendered socialization and the “thin-ideal” standards prevalent in contemporary society (Faragó et al., 2022). According to Social Learning Theory, individuals model the behaviors and attitudes of influential or admired figures (Bandura, 1977). In the digital age, ubiquitous access to celebrities and peers via social media fosters an environment of chronic social comparison, which may amplify the risk for AN symptoms among females (Mabe et al., 2014). This is consistent with empirical evidence demonstrating a robust correlation between appearance-based anxiety and intensive social media engagement (Papapanou et al., 2023).

From a developmental perspective, sociocultural pressures likely converge with early caregiving experiences to heighten the risk for AN symptoms in females. Research indicates that caregivers of female adolescents often place a greater emphasis on physical appearance and attire than those of male adolescents, inadvertently instilling a preoccupation with aesthetic standards from a young age (Dubovitskaya et al., 2020). As noted by Casale et al. (2025), when autonomy is restricted through overprotective or appearance-focused parenting, individuals may utilize restrictive eating as a compensatory domain where agency feels tangible. Thus, the modest moderation effect likely reflects the cumulative impact of these gendered socialization practices rather than a fundamental divergence in psychological architecture.

Finally, an alternative interpretation for this modest effect involves the issue of measurement invariance. It is possible that the psychometric properties of the instruments—specifically the ARSS and EDI subscales—vary across groups. If males and females interpret or respond to items regarding “body dissatisfaction” or “rejection anxiety” differently, the observed interaction might partially reflect measurement artifacts rather than true psychological variance. Future research should employ rigorous multigroup invariance testing to further clarify these gendered nuances.

### 4.3. Limitations and Future Direction

This study explored how attachment anxiety is associated with AN symptoms through ARS, with females experiencing a stronger effect in this process. However, there are several limitations that should be emphasized.

First, in a society where interpersonal relationships are increasing at unprecedented rates, individuals with anxious attachment may experience greater stress due to preoccupations with how others view them. As noted earlier, contemporary media facilitates increased access to images of others’ slender figures and desirable appearances. Previous studies have shown that these representations can elicit social comparisons related to physical appearance, contributing to appearance anxiety and lower body satisfaction, particularly among females (Seekis & Kennedy, 2023). The present study found that attachment anxiety can contribute to AN symptoms through the mediating role of ARS, and based on the above research, future research could further explore the impact of social comparison and online information on this process.

Second, this study used cross-sectional data, limiting our ability to infer causality. While the moderated mediation model is based on the theoretical framework of attachment theory, CSLT, and SLT, suggesting that attachment anxiety contributes to AN symptoms, it is possible that high levels of AN symptoms could drive increased attachment anxiety, particularly regarding others’ opinions. Future research should employ randomized controlled trials or longitudinal designs to test the causal relationships between these variables.

Third, the study population is restricted to Chinese university students, which limits the external validity of the findings. University students represent a relatively homogeneous group with distinct characteristics such as similar educational backgrounds and concentrated living environments. Their psychological experiences and life contexts differ from other populations such as high school students, young adults in the workplace, or community-dwelling individuals with diverse life trajectories. Moreover, the conclusions of this study are based on data obtained from a non-clinical population. Thus, while attachment anxiety was identified as a risk factor for subclinical AN symptoms, this does not imply that attachment anxiety causes clinically diagnosed AN. Future studies should expand the sample to include adolescents, young adults in non-academic settings, and clinically diagnosed patients with eating disorders.

Fourth, a notable limitation is that gender was operationalized using a binary framework (male vs. female) in the current study. This approach fails to capture the full spectrum of gender identity, thereby excluding the experiences of non-binary, gender-fluid, or gender-diverse individuals. Given that these populations often face unique stressors related to attachment, appearance-based rejection, and disordered eating, treating gender as a binary variable may oversimplify the complex interplay between identity and mental health. Consequently, the generalizability of our moderated mediation model to gender-diverse populations remains unknown. Future research should employ more inclusive, multi-dimensional measures of gender identity to provide a more comprehensive and representative understanding of these psychological pathways.

Furthermore, although attachment anxiety is theoretically rooted in early caregiving, our use of the RAAS self-report measure captures only current relational representations rather than objective historical facts. Consequently, the cross-sectional design of this study precludes any causal inferences regarding the developmental origins of these attachment patterns. Future research employing longitudinal tracking or retrospective clinical interviews is necessary to substantiate the specific developmental trajectory from early caregiving experiences to the emergence of anorexia symptoms in adulthood.

Finally, it is undeniable that not all individuals conform to sex stereotypes from social norms. Future research could explore personality differences rather than sex diversity, such as the Big Five personality traits, to provide a more nuanced understanding of the association between attachment anxiety and AN symptoms (Ulu & Tezer, 2010). For example, individuals with high attachment anxiety and high neuroticism may exhibit more AN symptoms compared to those with low neuroticism.

### 4.4. Implications

Despite its limitations, this study provides valuable insights into vulnerability markers for subclinical AN symptoms among college students. First, the findings emphasize the significant link between attachment anxiety and AN symptoms. Based on this, psychologists should increase attention to attachment styles, family functioning, parenting styles, and communication patterns when developing interventions to mitigate early vulnerability to AN symptoms, particularly in educational settings (Dring, 2015). Second, this study advances the understanding of the moderating role of sex in the relationship between attachment anxiety and AN symptoms, indicating that ARS may cause greater psychological harm to females. Some researchers have found that psychological sex differences are smaller in nations with greater sex equality and larger in nations with greater inequality (Hyde, 2014). Therefore, promoting social equity to mitigate the harmful effects of appearance-based social comparisons on females may be an effective strategy. Future research could use laboratory studies to create different social equity scenarios and examine the negative impact of ARS on females. In summary, the findings of the present study provide empirical evidence and theoretical support for future research, particularly studies exploring body image-related societal pressures, gender-specific psychological mechanisms, and the design of targeted interventions aimed at fostering gender equality and enhancing overall well-being.

## 5. Conclusions

This study examined the relationship between attachment anxiety and subclinical AN symptoms among Chinese college students and investigated the mediating effect of appearance-based rejection sensitivity and the moderating effect of sex in this relationship. The results showed that attachment anxiety was positively correlated with AN symptoms, and ARS mediated this relationship. Moreover, AN symptoms and ARS were more severe in females, and the associations between these variables were stronger in females. These findings provide theoretical and empirical support for psychological interventions to reduce AN symptoms, especially among individuals with attachment anxiety.

## Figures and Tables

**Table 1:** Demographic characteristic (*N* = 826).

Variables	Male	Female	χ^2^	*p*
Relationship status			2.33	0.13
Single	256 (0.78)	366 (0.73)		
In a relationship	71 (0.22)	133 (0.27)		
Romantic experiences			0.01	0.93
Yes	184 (0.56)	278 (0.56)		
No	143 (0.44)	221 (0.44)		
Subjective economic status			26.85	<0.001
Very low	56 (0.17)	39 (0.08)		
Low	102 (0.31)	125 (0.25)		
Medium	157 (0.48)	311 (0.62)		
High	10 (0.03)	23 (0.05)		
Very high	2 (0.01)	1 (<0.01)		
Father education level			7.06	0.20
Junior high and below	160 (0.49)	225 (0.45)		
Senior high	90 (0.28)	138 (0.28)		
Bachelor	53 (0.16)	112 (0.22)		
Master	11 (0.03)	10 (0.02)		
Doctor	4 (0.01)	3 (0.01)		
Reluctant to disclose	9 (0.03)	11 (0.02)		
Mother education level			11.20	0.03
Junior high and below	205 (0.63)	270 (0.54)		
Senior high	60 (0.18)	115 (0.23)		
Bachelor	47 (0.14)	100 (0.20)		
Master	3 (0.01)	2 (<0.01)		
Doctor	1 (<0.01)	0 (0.00)		
Reluctant to disclose	11 (0.03)	12 (0.02)		

**Table 2:** Descriptive statistics and *t*-test results.

Variable	M	SD	Skew	Kurtosis	Female M ± SD	Male M ± SD	t	Cohen’s d
AA	2.79	0.77	0.15	−0.18	2.82 ± 0.78	2.73 ± 0.76	1.63	0.12
ARS	8.42	5.17	1.14	1.90	8.83 ± 5.15	7.80 ± 5.16	2.83 **	0.20
AN	3.19	0.91	0.13	−0.59	3.45 ± 0.86	2.80 ± 0.84	10.74 ***	0.76

Note: ** *p* < 0.01. *** *p* < 0.001. AA = Attachment Anxiety; ARS = Appearance-based Rejection Sensitivity; AN = Anorexia Nervosa symptoms.

**Table 3:** Pearson correlations among variables.

Variable	1	2	3	4	5	6	7	8	9
1. Age	-								
2. Sex	0.07 *	-							
3. Relationship status	0.12 ***	0.05	-						
4. Romantic experiences	−0.06	0.01	−0.42 ***	-					
5. SES	−0.07	0.16 ***	0.05	−0.11 **	-				
6. Father education	−0.22 ***	0.01	0.07 *	−0.04	0.33 ***	-			
7. Mother education	−0.21 ***	0.04	0.04	−0.01	0.32 ***	0.80 ***	-		
8. Attachment anxiety	−0.04	0.06	−0.13 ***	0.09 **	−0.08 *	−0.06	−0.04	-	
9. ARS	0.04	0.35 ***	−0.07	0.07	0.07 *	0.05	0.08	0.26 ***	-
10. AN	0.02	0.10 **	−0.05	0.09 *	−0.02	−0.00	−0.00	0.40 ***	0.40 ***

Note: * *p* < 0.05. ** *p* < 0.01. *** *p* < 0.001. ARS = Appearance-based Rejection Sensitivity; AN = Anorexia Nervosa symptoms; Sex: 1 = male, 2 = female; Relationship status: 1 = single, 2 = in a relationship; Romantic experiences: 1 = yes, 2 = no; SES = Subjective Economic Status: 1 = very low, 2 = low, 3 = medium, 4 = high, 5 = very high; Father/Mother education: 1 = junior high and below, 2 = senior high, 3 = bachelor, 4 = master, 5 = doctor.

**Table 4:** Fitting results of the mediation model and the moderated mediation model (N = 826).

Process	Predictors	Mediation Model				Moderated Mediation Model			
		b	SE	t	β	b	SE	t	β
1. Mediator variable model (ARS)	Constant	−4.716	2.818	−1.673	−1.036	−4.716	2.818	−1.673	−1.036
	Age	0.159	0.136	1.171	0.040	0.159	0.136	1.171	0.040
	Relationship status	0.250	0.426	0.587	0.019	0.250	0.426	0.587	0.019
	Romantic experiences	0.659	0.369	1.786	0.063	0.659	0.369	1.786	0.063
	SES	0.088	0.232	0.377	0.013	0.088	0.232	0.377	0.013
	Father education	0.207	0.251	0.825	0.045	0.207	0.251	0.825	0.045
	Mother education	−0.101	0.256	−0.394	−0.021	−0.101	0.256	−0.394	−0.021
	Attachment anxiety	2.720	0.216	12.573 ***	0.406	2.720	0.216	12.573 ***	0.406
2. Dependent variable model (AN)	Constant	−1.205	0.491	−2.453 *	−1.435	−1.672	0.465	−3.597 ***	−1.876
	Age	0.049	0.024	2.070 *	0.065	0.037	0.022	1.669	0.043
	Relationship status	−0.079	0.074	−1.071	−0.039	−0.122	0.070	−1.735	−0.060
	Romantic experiences	0.038	0.064	0.585	0.021	0.009	0.061	0.152	0.005
	SES	0.088	0.044	2.182 *	0.074	0.023	0.039	0.599	0.020
	Father education	−0.023	0.018	−0.533	−0.027	−0.002	0.041	−0.041	−0.003
	Mother education	0.083	0.045	1.856	0.098	0.072	0.042	1.712	0.089
	Attachment anxiety	0.142	0.041	3.464 ***	0.121	0.125	0.039	3.217 **	0.111
	ARS	0.061	0.006	10.042 ***	0.347	0.017	0.006	3.00 **	0.102
	Sex					0.573	0.057	10.117 ***	0.323
	ARS × Sex					0.025	0.003	7.989 ***	0.247
		R^2^ = 0.188, F = 23.675, df_1_ = 8, df_2_ = 817				R^2^ = 0.282, F = 31.974, df_1_ = 10, df_2_ = 815			
Process				Condition		Effect	Bootstrapped SE	Bootstrapped 95% CI	
3. Conditional indirect effects of AA on AN through ARS according to sex				Male		0.114	0.031	[0.059, 0.182]	
				Female		0.181	0.024	[0.137, 0.230]	

Note: * *p* < 0.05. ** *p* < 0.01. *** *p* < 0.001. ARS = Appearance-based Rejection Sensitivity; AN = Anorexia Nervosa symptoms; SES = Subjective Economic Status. All βs are unstandardized coefficients.

## Data Availability

The data presented in this study are openly available in OSF at https://osf.io/87sjq/overview?view_only=98ff47f70b844d888f7acb79bedb6507 (accessed on 5 February 2026).
